# Characterization of ecto- and endoparasite communities of wild Mediterranean teleosts by a metabarcoding approach

**DOI:** 10.1371/journal.pone.0221475

**Published:** 2019-09-10

**Authors:** Mathilde Scheifler, Magdalena Ruiz-Rodríguez, Sophie Sanchez-Brosseau, Elodie Magnanou, Marcelino T. Suzuki, Nyree West, Sébastien Duperron, Yves Desdevises

**Affiliations:** 1 Sorbonne Université, CNRS, Biologie Intégrative des Organismes Marins, BIOM, Observatoire Océanologique, Banyuls/Mer, France; 2 Sorbonne Université, CNRS, Laboratoire de Biodiversité et Biotechnologies Microbiennes, LBBM Observatoire Océanologique, Banyuls/Mer, France; 3 Sorbonne Université, CNRS, Observatoire Océanologique de Banyuls, Banyuls/Mer, France; 4 CNRS, Muséum National d’Histoire Naturelle, Molécules de Communication et Adaptation des Micro-organismes, UMR7245 MCAM, Muséum National d’Histoire Naturelle, Paris, France; Lund University, SWEDEN

## Abstract

Next‐generation sequencing methods are increasingly used to identify eukaryotic, unicellular and multicellular symbiont communities within hosts. In this study, we analyzed the non-specific reads obtained during a metabarcoding survey of the bacterial communities associated to three different tissues collected from 13 wild Mediterranean teleost fish species. In total, 30 eukaryotic genera were identified as putative parasites of teleosts, associated to skin mucus, gills mucus and intestine: 2 ascomycetes, 4 arthropods, 2 cnidarians, 7 nematodes, 10 platyhelminthes, 4 apicomplexans, 1 ciliate as well as one order in dinoflagellates (Syndiniales). These results highlighted that (1) the metabarcoding approach was able to uncover a large spectrum of symbiotic organisms associated to the fish species studied, (2) symbionts not yet identified in several teleost species were putatively present, (3) the parasitic diversity differed markedly across host species and (4) in most cases, the distribution of known parasitic genera within tissues is in accordance with the literature. The current work illustrates the large insights that can be gained by making maximum use of data from a metabarcoding approach.

## Introduction

Parasites are extremely diverse and omnipresent in all environments, making this lifestyle one of the most successful on Earth [1]. Parasites are usually classified as ectoparasites or endoparasites, with or without direct contact with the external environment respectively [2]. Parasites also differ in their life cycle: some parasites require only one host to complete their life cycle (direct life cycle) while others need intermediate hosts (indirect life cycle), in which they change their morphology and biology (mobility, reproduction, …) [2]. Fish are well known to be parasitized by many eukaryotic organisms, unicellular or multicellular [3–6]. Fish gills and skin, constantly in contact with the surrounding water, are exposed to ectoparasitic organisms. The mucus layer covering skin and gills might act as a first barrier against these putative pathogenic organisms [7]. Endoparasites colonize the internal organs or tissues of their hosts, such as the thoracic cavity, muscles or organs of the digestive and urinary tract [2]. Through their impacts on host fitness and on host interactions with the environment (competition, predation, behavioral changes), parasites play an essential role in population dynamics and ecosystems functioning [1,8–10].

Studying parasitological communities is tedious, as traditional surveys involve lots of steps and deep knowledge of often small and complex organisms. Traditionally, parasitological surveys are performed by host dissection to recover parasites, followed by identification under a microscope based on morphological criteria, mostly of the adult forms [11]. The morphological identification of parasites requires extensive taxonomic expertise and is very time consuming [12–13]. It is then difficult to perform exhaustive studies of parasitic communities considering all different hosts and all parasite life stages based solely on morphology (many parasites being of very small size or even microscopic). A lot of parasites, such as helminths or copepods, go through different life stages (i.e. cyst, egg and larval stages) [14–17] that are often difficult or nearly impossible to locate [18] and even if some of them can be detected, morphological differences are insufficient to differentiate between species from these development stages [19–20]. In addition, these surveys usually focus on a single class of parasites, ecto- or endoparasites [21–24] and sometimes even on specific genera [25–27]. However, new sequencing methods have emerged as having the potential to more effectively characterize parasitic communities and open new avenues for parasitological research.

The use of high-throughput sequencing methods, such as metabarcoding is now commonplace in many fields of biological research [28–30]. This technique has already revolutionized our understanding of microbial diversity [31–33] and its use is booming in the field of eukaryotic diversity [12–13,34–35]. Metabarcoding allows a more in-depth investigation of entire parasite communities [36–39], including all life stages. Tanaka *et al*. (2014) compared the helminthic diversity of wild rat intestines using a traditional and a metabarcoding approach. They highlighted that the metabarcoding approach is reliable and useful to investigate parasitic diversity and should allow more accurate identification of parasites than a traditional method. In assessing the number of reads per parasitic species, they also hypothesized about the life stage of several parasitic species present (eggs, sexually immature adults, …) [37]. Moreover, this technique is faster than traditional methods, relatively easier to perform and sensitive enough to be performed in a high-throughput manner [11,40–41].

In this study, we characterized the parasitic communities associated to skin mucus, gill mucus and intestine of several wild Mediterranean teleost fish species, by analyzing the non-specific reads obtained during a metabarcoding survey of the bacterial communities. In a global study of fish associated microbiota [42], we employed 16S rDNA primers biased towards bacteria and archaea [43]. However, these primers that can also amplify the 18S rDNA from eukaryotic organisms [43–44], also yielded several thousand reads that corresponded to eukaryotes. We further investigated the identity of these eukaryotic sequences associated with skin mucus, gill mucus and intestine of several teleost fish species from the Bay of Banyuls-sur-Mer (Gulf of Lion, northwest Mediterranean, France). We focused on 13 species, representing 5 families, easily observable in the Mediterranean Sea and each presenting different lifestyles, behavior and diet. Our aims were to i) test whether our metabarcoding approach, initially targeting bacterial DNA, was able to detect, in addition to bacterial communities, a symbiotic eukaryotic community present on the host and ii) assess whether they could correspond to previously undetected parasites, compared to what is known in the literature. Eukaryotic sequences obtained in initially bacteria-targeting metabarcoding approaches could be a cheap way to gain at least some knowledge about the hidden diversity of eukaryotic parasites.

## Materials and methods

### Ethics statement

The Observatoire Océanologique de Banyuls-sur-Mer holds the authorization for fishing and housing wild Mediterranean teleosts (Decision n°100/2019, Direction Interrégionale de la Mer Méditerranée). Wild fish were caught (see below for details) by a competent person and in accordance with the European Union Regulations concerning the protection and welfare of experimental animals (European directive 91/492/CCE).

### Fish sampling

Thirteen teleost fish species were collected in June 2017 (42°29’4.618”N, 3°8’,35.39”E) and in October 2017 (42°29’15.073”N, 3°7’,49.688”E) in the Bay of Banyuls-sur-Mer (northwest Mediterranean, France) (Table 1). Between two and five teleost specimens were collected for each species (Table 1). A gill fishing net was placed overnight in Banyuls bay between 0 and 6 m deep. About 6 hours later, fish were collected dead on the net, handled with gloves and put into individual plastic bags right after collection from the net. They were immediately brought from the vessel to the laboratory for dissection. Three different tissues were collected per fish individual and put into sterile tubes: skin mucus, gill mucus (with two gill arches) were collected with a sterile spatula and scissors, and for species that did not have mucus on their skin, we collected a portion of skin close to the lateral line of the fish (around 3 cm^2^) by using ethanol-rinsed scissors and tweezers; a fragment (between 3 and 5 cm long) of the posterior region of the intestine was also sampled (depending on the size of fish specimens). Samples were frozen at -80°C until DNA extraction.

**Table 1:** Number of samples collected for each fish species.

		Samples				
	Fish family	Skin mucus	Skin	Gill mucus	Intestine	
*Diplodus annularis*^*1*^	Sparidae	5		5	3	
*Diplodus vulgaris*^*4*^	Sparidae	2		5	2	
*Gobius bucchichi*^*1*^^,^^*2*^^,^^*3*^	Gobiidae	3		5	3	
*Gobius cruentatus*^*2*^	Gobiidae	2		2	2	
*Gobius niger*^*3*^^,^^*4*^	Gobiidae	2		3	3	
*Oblada melanura*^*1*^^,^^*4*^	Sparidae	4		5	3	
*Pagellus bogaraveo*^*1*^	Sparidae	3		4	2	
*Pagellus erythrinus*^*1*^	Sparidae	5		5	5	
*Sarpa salpa*^*4*^	Sparidae	5		5	3	
*Serranus scriba*^*1*^	Serranidae	5		5	3	
*Scorpaena notata*^*1*^	Scorpaenidae		5	5	4	
*Spicara maena*^*1*^	Sparidae	4		4	2	
*Symphodus tinca*^*1*^	Labridae	5		5	3	
*Total*		45	5	58	38	146

Sampling in 2017

^1^June 21^th^

^2^June 26^th^

^3^July 18^th^ and

^4^October 4^th^.

As mentioned before, aquatic organisms are constantly in contact with the surrounding water. In skin mucus, gill mucus and intestine, not only symbionts and DNA from hosts were recovered but also what is present in the surrounding environment (food and free-living organisms) and most likely traces of DNA leaved by all organisms, reflecting their current or past presence (i.e. environmental DNA) [34]. In order to verify that our sampling techniques were appropriate for the identification of parasitic taxa specifically associated to fish species (i.e. not present in the surrounding water), seawater was collected using a sterile container at each sampling date next to the gill net fishing to act as negative controls. Two liters of seawater were filtered onto a 0.2 μm nitrocellulose filter (Pall Corporation, U.S.A). Filters of each sampling date were frozen at -80°C until DNA extraction.

### DNA extraction and amplification

DNA was extracted by using the Quick-DNA Fecal/Soil Microbe MiniPrep Kit (Zymo Research, Orange, California) following manufacturer’s instructions. Samples were frozen at -80°C. PCR amplification was carried out in triplicate and performed using primers targeting the hypervariable V4-V5 region of the 16S rRNA gene: 515F-Y (5’-GTGYCAGCMGCCGCGGTAA) and 926R (5’-CCGYCAATTYMTTTRAGTTT) [43]. The PCR mix contained 1X KAPA 2G Fast Ready Mix (Sigma-Aldrich, France), 0.2 μl of each primer (concentration of 0.2 μM), 3.6 μl of ultrapure water and 1 μl of DNA in a final volume of 10 μl. After 3 min of initial denaturation at 95°C, the following conditions were applied: 22 cycles of 95°C for 45s (denaturation), 50°C for 45s (annealing) and 68°C for 90s (extension), with a final extension at 68°C for 5 min. For each sample, three PCRs were performed in the same conditions, to increase the DNA quantity, but also to avoid bias due to each reaction. Then, the product of each PCR was run on a 1% agarose gel at 100V for 20 minutes in an electrophoresis chamber (BIO-RAD) to visualize the presence of high molecular weight DNA. The visualization was carried out in a GelMaxTM photodocumenter (UVP^®^). When the DNA was visible in the gel, amplifications from the same sample were pooled. Individual barcode sequences were added to each mix during a second PCR. The second PCR mix contained 1X KAPA 2G Fast Ready Mix (Sigma-Aldrich, France), 0.5 μl of each barcode (Nextera Index Sequences in http://seq.liai.org/204-2/), 10.5 μl ultrapure water and 1 μl of DNA for a final volume of 25 μl. PCR conditions were as follows: initial denaturation at 98°C for 30s, 8 cycles of 98°C for 10s, 60°C for 20s, 72°C for 30s and a final extension at 72°C for 2 min. Incubation (37°C for 30min, 85°C for 15 min) with USB ExoSAP-IT PCR Product Cleanup (Thermofisher, France) was then performed to degrade unincorporated primers. All PCR products were normalized with a 96 well SequalPrep Normalization Plate (Thermofisher, France) and the normalized amplicons were concentrated by using the Wizard SV Gel and PCR Clean up Kit (Promega, France) (final concentration around 8 ng/μl). Finally, the concentration of each DNA sample was measured using the Quant-iT^TM^ PicoGreen (Thermofisher, France) and amplicons were sequenced using Illumina 2×250 MiSeq sequencing (FASTERIS SA, Switzerland). Samples were run in two sessions and divided randomly. All the categories (fish species and body parts) were represented in both runs.

### Sequence analyses

Sequence analysis was realized using an in-house pipeline based on Needham *et al*., 2018 [44] and scripts in both Usearch9 [45] and Qiime V.1.9.1 [46]. A shell script 18Sclean.sh (see S1 Text) was used to pre-treat demultiplexed forward and reverse reads. Basically, as Needham and collaborators, we used the strategy of using non-overlapping reads to identify eukaryotic sequences among sequences amplified with the Parada and collaborator's primers [43]. Unassembled reads, identified using PEAR v.0.9.6 [47], were quality trimmed using the *fastq_filter* option of *usearch9*. Sequences in fasta format with both reads for each sample were merged with an "N" between the forward and the reverse-complemented reads. Sequences names were modified to include a sample label. Samples from all reads were merged in a single fasta file. At this stage, primer sequences were removed and the reads were dereplicated. The number of singletons was determined and they were denoised using *usearch9 -unoise* with minsize = 1. Since denoising of concatenated sequences appear to yield a large proportion of singletons, denoised sequences were clustered in Operational Taxonomic Units (OTUs) using *usearch9 -cluster_otus* using a stringent radius of 0.75 and minsize = 1. OTU tables were generated by *usearch9 -usearch_global* using -id = 0.97.

All OTUs were identified using QIIME's *assign_taxonomy*.*py -m rdp* and the "SILVA_132_ QIIME_release/taxonomy/18S_only/99/majority_taxonomy_7_levels.txt" file distributed by the SILVA project [48–49]. Finally, this OTU table was filtered to remove fish (“D_12__Teleostei”) OTUs. Some sequences were assigned to taxonomic groups but not to a specific genus due to the poor representation of eukaryotic symbionts within the SILVA database [50–51]. To alleviate this problem, we used the program Basic Local Alignment Search Tool (BLAST, [52]) to assign them to a described genus whenever possible. Sequences with more than 98% similarity to a genus were kept and assigned. If a sequence matched with two or more genera, we used the OTU classification to choose the appropriate genus (with at least 98% similarity). In the absence of support from the OTU classification, or in the case of less than 98% similarity, the sequences were not assigned more precisely and not used in the analysis. Finally, all sequences unassigned to any taxonomic group (only 0.4% of all reads) were excluded. The remaining OTUs were used to assess the total eukaryotic diversity.

## Results

A total of 146 samples were collected from 59 fish specimens in the Bay of Banyuls-sur-Mer (Table 1). The species *Scorpaena notata* did not have any mucus on its skin, so we collected a portion of the skin. The skin was treated as skin mucus for further analyses. A total of 3,064,621 sequences of minimum 400 bp length were obtained for the 146 samples. Around 76.2% of these reads corresponded to Bacteria and Archaea, whereas the rest of the sequences were assigned as eukaryotes. We investigated 44,258 sequences in total (representing 6.1% of eukaryotic reads) as potentially fish-associated symbionts (mutualistic, parasitic and commensal eukaryotic organisms; 93.8% of eukaryotic reads are fish sequences). The mean number of reads of potential symbionts per sample is 303.2 (standard deviation = 591.9).

**Fig 1:**
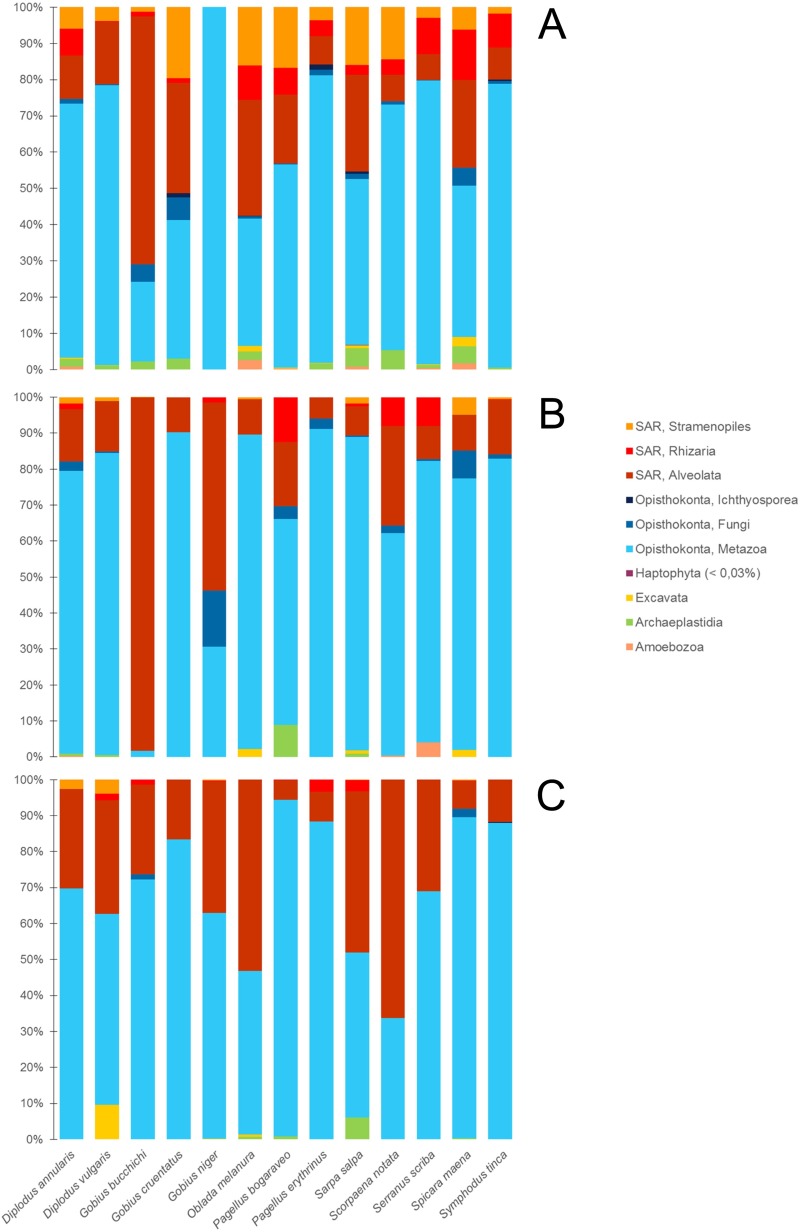
**Relative abundance of eukaryotic supergroups amoung all eukaryotic reads within each fish species in (A) skin mucus, (B) gills mucus and (C) intestine.** The number of specimens for each column for each tissue is indicated as follows: number of specimens of (skin mucus, gill mucus, intestine), *Diplodus annularis* (5,5,3), *D*. *vulgaris* (2,5,2), *Gobius bucchichi* (3,5,3), *G*. *cruentatus* (2,2,2), *G*. *niger* (2,3,3), *Oblada melanura* (4,5,3), *Pagellus bogaraveo* (3,4,2), *P*. *erythrinus* (5,5,5), *Sarpa salpa* (5,5,3), *Scorpaena notata* (5,5,4), *Serranus scriba* (5,5,3), *Spicara maena* (5,5,2), *Symphodus tinca* (5,5,3) (see Table 1).

For each species and for each tissue, we computed the mean value of the proportions in the samples. All sequences assigned to fish were removed. Among Eukaryota, representatives of clades Opisthokonta and SAR (Stramenopiles, Alveolates and Rhizaria) were detected in all tissues (skin mucus, gills mucus and intestine) of all the fish species. They were the most abundant groups, representing 88.16 to 100% of eukaryote-affiliated reads (Fig 1). The opisthokonts are a broad group of eukaryotes, composed of fungi, Ichthyosporea and metazoans. All of these three groups are represented in our analyses, but metazoans are ubiquitous and the most abundant in most samples (Fig 1). Within the SAR group, alveolates were detected in all tissues. The supergroups Stramenopiles, Rhizaria, Fungi and Ichthyosporea were mainly present on the external surfaces of fish (13.95% and 5.06% on average for skin mucus and gill mucus respectively compared to 1.53% for intestine) (Fig 1). The four other supergroups, Haptophyta, Excavata, Archeaplastidia and Amoebozoa together accounted for < 10% of the sequences in skin mucus and < 9% of the sequences in gill mucus. They were mostly absent from the intestinal samples, apart from supergroup Archaeplastida retrieved from *Salpa salpa*, which represented 6.1% of eukaryotic sequences from this fish species (Fig 1C).

As this study focuses on parasitic communities of teleost fish species, sequences belonging to non-parasitic organisms were removed from the analysis. Therefore, we have eliminated most of photosynthetic organisms (i.e. green algae, red algae and green plants (Archaeplastida), some diatoms and dinoflagellates) [53], common dietary items and free-living organisms. Parasites likely coming from organisms eaten by fish were also discarded (such as from, very likely, plant or arthropod hosts). In order to determine parasitic genera associated to fish, bibliographic searches were carried out using PubMed, Google Scholar and google searches. For each parasitic genus, we used keywords: "parasite", "fish" / "fish species", "name of the parasitic genus". Finally, only parasitic genera that were present at least in two samples (among the 146 collected) and with a minimum of 10 reads were retained for further examination (S1 Table).

We identified 30 genera as potential parasites of teleosts, representing 24,274 reads (0.8% of total reads): 2 Ascomycota, 4 Arthropoda, 2 Cnidaria, 7 Nematoda, 10 Platyhelminthes, 4 Apicomplexa, and 1 Ciliophora (Table 2). None of these parasitic genera has been identified in water samples (S2 Table).

**Table 2:** Presence (black square) or absence (white square) of eukaryotic parasitic taxa within fish teleost species. The black squares with white diagonals indicate fish-parasite associations already listed in the literature (S2 Text). References confirm the parasitic status of the genera. Fish species: ^1^*Diplodus annularis*, ^2^*Diplodus* vulgaris, ^3^*Gobius bucchichi*, ^4^*Gobius cruentatus*, ^5^*Gobius niger*, ^6^*Oblada melanura*, ^7^*Pagellus bogaraveo*, ^8^*Pagellus erythrinus*, ^9^*Sarpa salpa*, ^10^*Scorpaena notata*, ^11^*Serranus* scriba, ^12^*Spicara maena*, ^13^*Symphodus tinca*.

		Fish species												
		1	2	3	4	5	6	7	8	9	10	11	12	13
Phylum, Class	Genus													
Ascomycota, Eurotiomycetes	*Aspergillus* [54–55]													
Ascomycota, Dothideomycetes	*Cladosporium* [56]													
Arthropoda, Copepoda	*Caligus* [57–58]													
Arthropoda, Copepoda	*Chondracanthus* [59–60]													
Arthropoda, Copepoda	*Lepeophtheirus* [61–62]													
Arthropoda, Copepoda	*Taeniacanthus* [63–64]													
Cnidaria, Myxozoa	*Kudoa* [65]													
Cnidaria, Myxozoa	*Unicapsula* [66–67]													
Nematoda, Chromadorea	*Acanthocheilus* [68]													
Nematoda, Chromadorea	*Contracaecum* [69–70]													
Nematoda, Chromadorea	*Cucullanus* [27,71]													
Nematoda, Chromadorea	*Dichelyne* [26,72]													
Nematoda, Chromadorea	*Cystidicola* [73–74]													
Nematoda, Chromadorea	*Hysterothylacium* [75–76]													
Nematoda, Enoplea	*Aonchotheca* [77–78]													
Platyhelminthes, Monogenea	*Lamellodiscus* [25,79]													
Platyhelminthes, Monogenea	*Microcotyle* [80–81]													
Platyhelminthes, Monogenea	*Polylabris* [82–83]													
Platyhelminthes, Digenea	*Cardiocephaloides* [84–85]													
Platyhelminthes, Digenea	*Skoulekia* [86–87]													
Platyhelminthes, Digenea	*Accacoelium* [88]													
Platyhelminthes, Digenea	*Rhipidocotyle* [89–90]													
Platyhelminthes, Digenea	*Lecithochirium* [91–92]													
Platyhelminthes, Digenea	*Opisthorchis* [59,93]													
Platyhelminthes, Digenea	*Diphtherostomum* [94–95]													
Apicomplexa, Acanoidasida	*Theileria* [96–97]													
Apicomplexa, Canoidasida	*Cryptosporidium* [98–99]													
Apicomplexa, Canoidasida	*Eimeria* [100–101]													
Apicomplexa, Canoidasida	*Goussia* [102–103]													
Ciliophora, Oligohymenophorea	*Trichodina* [104–105]													
Dinoflagellata, Syndiniophyceae	Order Syndiniales [106]													
	**Total number of parasitic taxa**	**9**	**12**	**12**	**6**	**11**	**16**	**10**	**14**	**16**	**11**	**7**	**7**	**17**

Despite the fact that no genus could be determined within the order Syndiniales in our study, this order was also retained because some species are known to parasitize fish [106].

**Fig 2:**
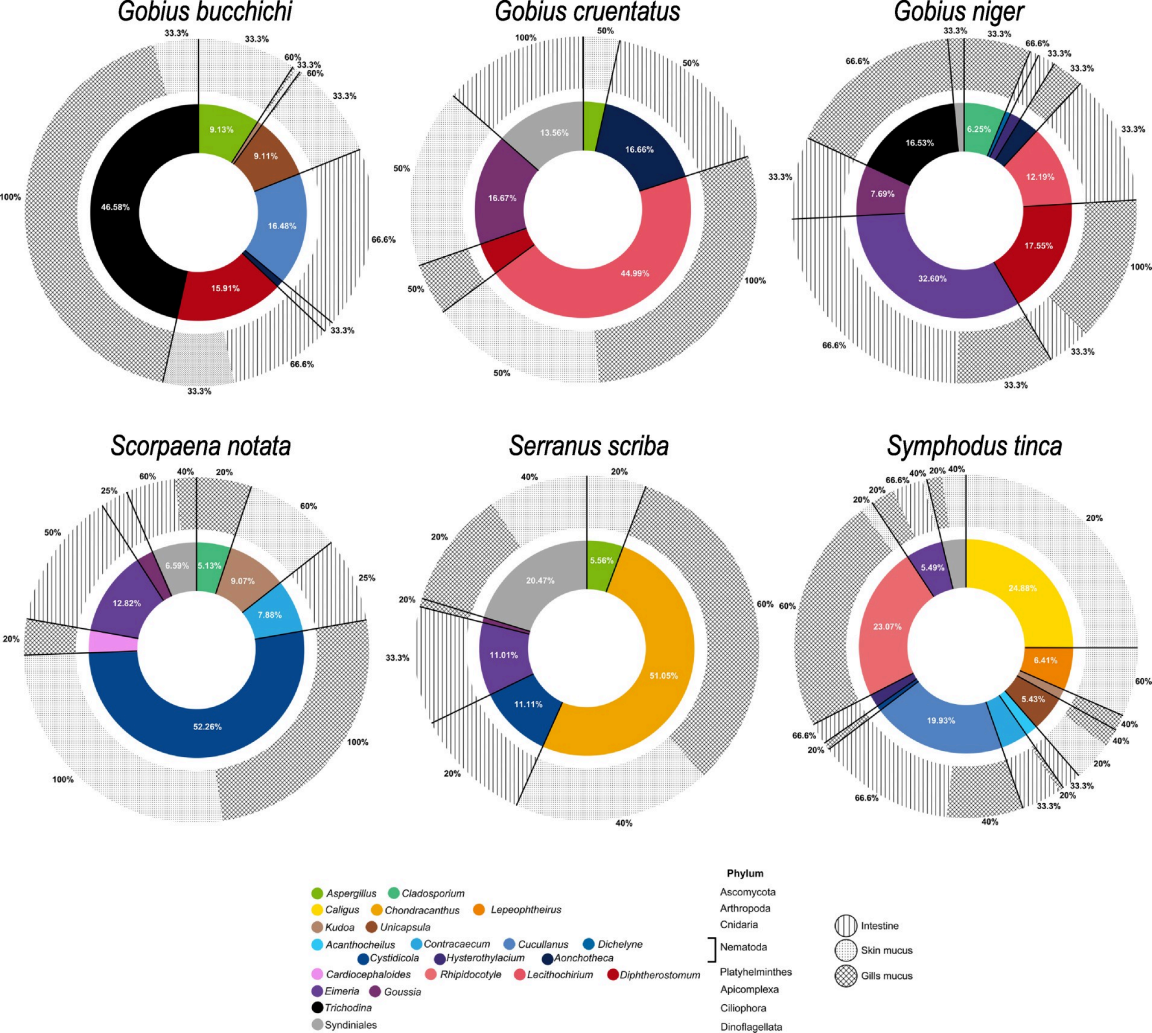
Eukaryotic parasitic community structure and distribution of each parasitic taxa within skin mucus, gill mucus and intestine for the fish family Sparidae. Percentages of parasitic taxa are indicated for proportions greater than 5%. Prevalence (proportion of individuals infected by a parasite taxon) is provided as a percentage around the outer circle.

**Fig 3:**
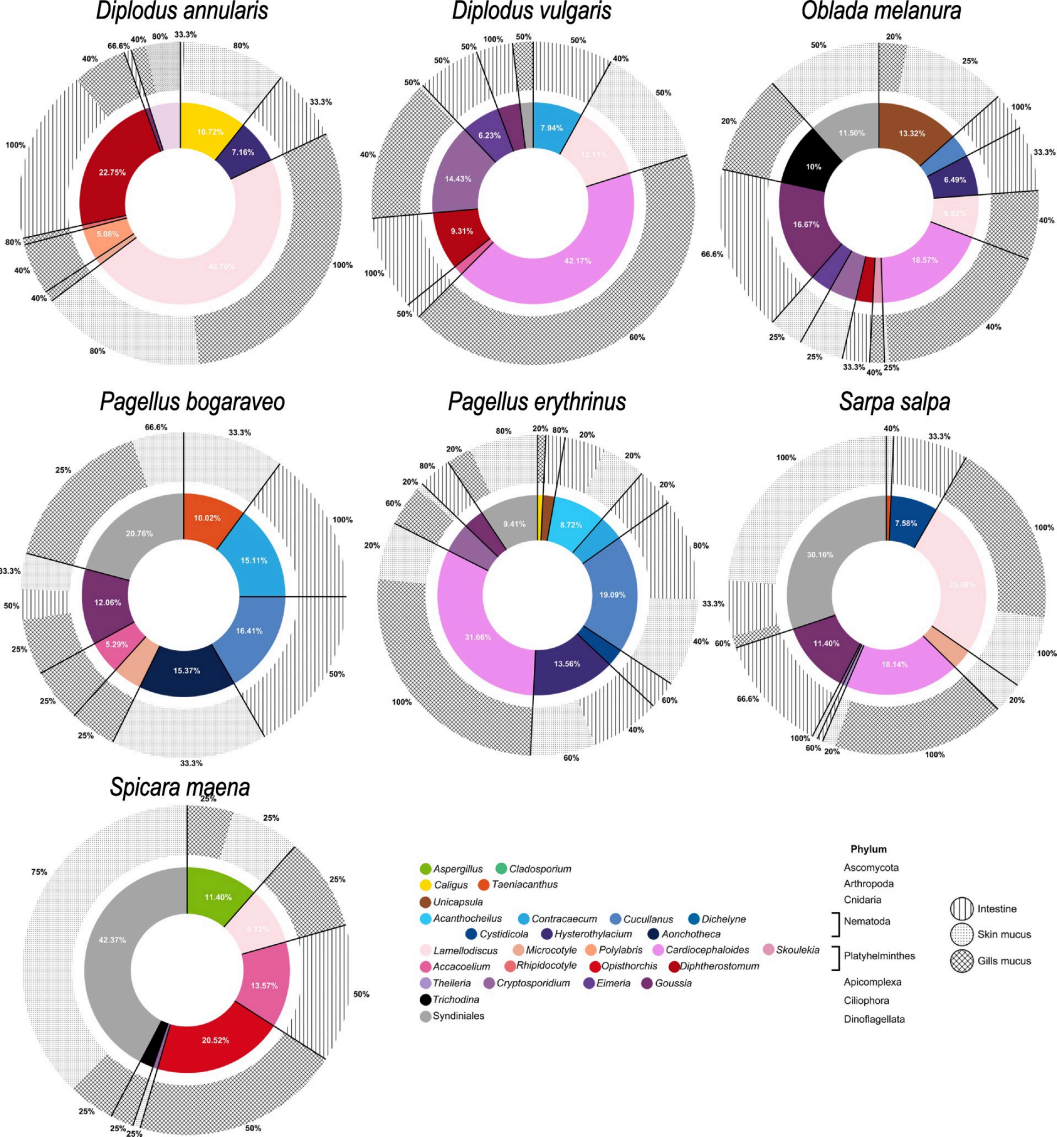
Eukaryotic parasitic community structure and distribution of each parasitic taxa within skin mucus, gills mucus and intestine for the fish families Gobiidae, Scorpaenidae, Serranidae and Labridae. Percentages of parasitic taxa are indicated for proportions greater than 5%. Prevalence (proportion of individuals infected by a parasite taxon) is provided as a percentage around the outer circle.

The structure of the parasitic communities was further investigated for each fish species (Figs 2 and 3). For greater clarity, only parasitic taxa representing at least 0.5% of eukaryote reads (obtained for each species) were represented (Figs 2 and 3, Table 2). All fish species were associated to several parasitic genera but their distribution greatly differed among host species (Figs 2 and 3). The genera *Aspergillus* (Ascomycota), *Eimeria* (Apicomplexa) and *Goussia* (Apicomplexa), as well as the order Syndiniales (Dinoflagellata) were ubiquitous in all the analyzed teleost families (Figs 2 and 3, Table 2). However, some taxa seemed to be more group specific to some hosts. For example, sequences from *Chondracanthus* (Copepoda) were detected only on *Serranus scriba* (51.1% of putative parasitic sequences identified on *Serranus scriba*, Fig 3E). Similarly, sequences from *Lepeophtheirus* (Copepoda) and *Polylabris* (Platyhelminthes, Monogenea) were only found in *Symphodus tinca* (6.4%, Fig 3F) and *Diplodus annularis* (5.1%, Fig 2A) respectively. Moreover, 1625 reads (6.7% of potential sequences from parasites) belonging to the genera *Taeniacanthus* (Copepoda), *Lamellodiscus*, *Microcotyle*, *Polylabris* (Monogenea) and *Opisthorchis* (Digenea) were only detected within the fish family Sparidae (Fig 2). No parasitic genus was found specifically associated to the Gobiidae family.

Among the 31 parasitic taxa, 9 genera (*Caligus* (Copepoda), *Unicapsula* (Myxozoa), *Cystidicola* and *Aonchotheca* (Nematoda), *Accacoelium*, *Lecithochirium* (Platyhelminthes), *Theileria*, *Eimeria and Goussia* (Apicomplexa)) and the order Syndiniales were observed in all tissues (gills mucus, skin mucus and intestine). Only 4 genera were present in only one tissue: *Lepeophtheirus*, *Taeniacanthus* (Copepoda) and *Polylabris* (Monogenea) were only observed in skin mucus, whereas *Opisthorchis* (Digenea) was only associated to fish gills. The other 17 genera were detected in two tissues (Table 3). Overall, sequences from 4 supergroups were mainly found in skin mucus: Ascomycota (65.5% of sequences), Arthropoda (82.1%), Cnidaria (93.9%) and Dinoflagellata (91.6%) (S3 Table). Nematoda and Apicomplexa were mainly identified in fish intestine (85.7% and 77.8% respectively) whereas Platyhelminthes and Ciliophora in fish gills (S3 Table). Monogeneans and ciliates are the only taxa present in only two tissues (62.9% and 90.9% in gills mucus and 37.1% and 2.4% in skin mucus for monogeneans and ciliates, respectively) (S3 Table).

**Table 3:** Distribution of the 31 parasitic taxa within host gills mucus, skin mucus and intestine.

				Origin of reads (%)		
				Gills mucus	Skin mucus	Intestine
Phylum	Class	Genus	Number of reads			
Ascomycota	Eurotiomycetes	*Aspergillus*	13	15.4	**84.6**	0
Ascomycota	Dothideomycetes	*Cladosporium*	16	**50**	**50**	0
Arthropoda	Copepoda	*Caligus*	186	0.5	**95.7**	3.8
Arthropoda	Copepoda	*Chondracanthus*	43	**90.7**	9.3	0
Arthropoda	Copepoda	*Lepeophtheirus*	21	0	**100**	0
Arthropoda	Copepoda	*Taeniacanthus*	12	0	**100**	0
Cnidaria	Myxozoa	*Kudoa*	422	7.3	**92.7**	0
Cnidaria	Myxozoa	*Unicapsula*	949	0.1	**94.5**	5.4
Nematoda	Chromadorea	*Acanthocheilus*	3542	0	26.3	**73.7**
Nematoda	Chromadorea	*Contracaecum*	2893	10.2	0	**89.8**
Nematoda	Chromadorea	*Cucullanus*	922	2.1	0	**97.9**
Nematoda	Chromadorea	*Dichelyne*	4581	0	7.4	**92.6**
Nematoda	Chromadorea	*Cystidicola*	334	7.2	29.9	**62.9**
Nematoda	Chromadorea	*Hysterothylacium*	13	0	23.1	**76.9**
Nematoda	Enoplea	*Aonchotheca*	168	1.2	38.7	**60.1**
Platyhelminthes	Monogenea	*Lamellodiscus*	982	**72.1**	27.9	0
Platyhelminthes	Monogenea	*Microcotyle*	359	**83.6**	16.4	0
Platyhelminthes	Monogenea	*Polylabris*	262	0	**100**	0
Platyhelminthes	Digenea	*Cardiocephaloides*	856	**93.7**	0	6.3
Platyhelminthes	Digenea	*Skoulekia*	2401	**99.7**	0.3	0
Platyhelminthes	Digenea	*Accacoelium*	432	**99.1**	0.7	0.2
Platyhelminthes	Digenea	*Rhipidocotyle*	293	**98.3**	1.7	0
Platyhelminthes	Digenea	*Lecithochirium*	408	**74.8**	23.8	1.4
Platyhelminthes	Digenea	*Opisthorchis*	10	**100**	0	0
Platyhelminthes	Digenea	*Diphtherostomum*	246	0.8	0	**99.2**
Apicomplexa	Aconoidasida	*Theileria*	24	8.4	**45.8**	**45.8**
Apicomplexa	Conoidasida	*Cryptosporidium*	265	2.6	**97.4**	0
Apicomplexa	Conoidasida	*Eimeria*	361	3.9	1.1	**95**
Apicomplexa	Conoidasida	*Goussia*	834	3.7	0.4	**95.9**
Ciliophora	Oligohymenophorea	*Trichodina*	1972	**98.7**	1.3	0
Dinoflagellata	Syndiniophyceae		454	7.9	**91.6**	0.5

Each line represents the proportion of reads of the parasitic organism in each tissue in relation to the number of total eukaryotic reads obtained for this taxa. The tissue with the largest percentage of each genus is in bold.

## Discussion

In this study, we performed a detailed analysis of eukaryotic sequences that were recovered from a metabarcoding study that was initially targeting bacteria associated to different tissues of teleost fish. The approach indeed yielded around 44,258 eukaryotic sequences (around 1.4% of total reads) that potentially open a window to the diversity of parasites associated with fish species.

### Benefits of a metabarcoding approach

It is not surprising that we obtained many eukaryotic sequences with this primer set, from a metabarcoding approach initially targeting bacterial DNA. Indeed, the primer set was optimized to be universal for Bacteria and Archaea, but it has been also used to study the eukaryotic diversity, since in silico tests by Parada *et al*. 2016 [43] reportedly showed 0 mismatches to 86% of all eukaryotic sequences in the SILVA Database SSU r123 database. We repeated the analysis with 75,000 eukaryotic sequences present in the SILVA_132_SSURef_NR99_13_12_17_opt.arb tree of the Silva project [48–49] using the probe_match and the match function of arb_edit. The results show that only about 2200 sequences (c.a. 3%) had mismatches to the forward, and 4000 (c.a. 5%) to the reverse primer, with some groups having mismatches with both. Those mismatches were not against very broad groups; many mismatches were of a weak character (G-T mismatches) and in cases where a mismatch was to a broader group, these groups were not known to be marine, or associated with fish and thus we do not have any evidence that those primers would be particularly biased against a specific fish parasite group and invalidate our results. Moreover, the same primers have been also recently used to study symbiotic relationships including those of eukaryotes [44]. This approach gave us the opportunity to characterize, in addition to bacterial communities, the diversity of parasites associated with different fish tissues. These results highlight the fact that there are probably data on eukaryotic parasites in bacterial metabarcoding projects that are as yet unexploited.

Our results reveal diverse eukaryotic communities within several teleosts fish species that are far more taxonomically complex than anticipated by the current literature. This indicates that a metabarcoding approach allows for a faster and more complete identification of all parasites than traditional methods in parasitology. In the field of parasitology, description and identification of parasitic eukaryotic species are still dominated by morphological analyses [11]. Moreover, these studies generally focus on one or a few parasitic species of a specific phylum (e.g. arthropods, nematodes, copepods) [58,65,81,87,98]. In the past few years, gene metabarcoding approaches have been booming in the field of parasitology [37–38]. However, recent studies using this approach have focused mainly on characterizing a single compartment of biodiversity, including the nemabiome (or more broadly the helminthic community) of terrestrial species [39,107–109]. To our knowledge, this is the one of the first study to attempt to identify, without *a priori*, all parasitic eukaryotic taxa present on teleost fish species using a universal metabarcoding approach [110]. Even if this approach was initially intended to characterize the bacterial communities of some fish species, it has allowed us to detect parasitic species belonging to different phyla (Table 2) [29,35,41]. While inventories of parasitic eukaryotic species are generally limited to the study of macro-organisms or organisms visible with a stereomicroscope, the metabarcoding approach also provides access to a broader fraction of the parasitic diversity, including microorganisms, intracellular and intra-organellar parasites, and those that are difficult to identify using morphological criteria [29,39,111].

However, the metabarcoding approach applied to parasitic communities is still subject to limitations and some aspects need to be improved [39,108]. First, this approach allowed us to identify 24274 reads originating from potential parasites of teleosts among 146 samples. Some parasite genera have a large number of reads (*Acanthocheilus*, *Contracaecum*, …) while others have only a few dozen, such as *Aspergillus* or *Cladosporium* (Ascomycota). However, none of the parasitic genera identified in fish tissues were found in water samples. It reinforces the claim that there was no contamination among our samples and that the reads we identified do not correspond to eDNA [34] and are well associated with the different fish tissues. Even if this approach provides reliable information on the presence of parasitic species on a host organism [39,35,41], the presence of a species with a small number of reads should be taken cautiously. In addition, none of these data should be considered quantitative [112]. Parasitic load is defined as the number of parasites in a host individual and it can be measured directly by dissecting organisms to count the number of adult parasites of each species [113] or indirectly by quantifying the number of parasitic eggs in faeces, but this number is not necessarily related to the number of parasitic individuals in the host [39,114]. Moreover, in high-throughput sequencing technologies, many technical factors (DNA extraction, PCR primers suspected of amplifying the DNA of some species at the expense of others, and sequencing technique) but also biological factors (such as the amount of DNA that varies according to the species, size and stage of the individuals) can influence the number of times a sequence is observed [108,115]. Metabarcoding can assess a larger fraction of the biodiversity but abundances obtained from sequencing cannot be interpreted as with morphology-based methods, since it does not reflect quantitative data [13,112]. As is already the case in the microbial domain, methods must be developed to obtain and interpret quantitative data from high throughput sequencing of eukaryotic symbiotic communities [109,116]. In addition, high throughput sequencing may not be used to identify all species [35,41,107]. Indeed, reference databases for taxonomic assignment are mostly incomplete [50–51,117]. In this study, we used the SILVA 132 database, but despite regular updates [118], some sequences could not be assigned and, because they were in very low numbers, they were not included in our analysis. In the coming years, more taxa will be added to databases, allowing the scientific community to use the full potential of metabarcoding approaches to characterize broader symbiotic communities [107,119–120].

### Distribution of potentially parasitic genera

In this study, 30 genera (distributed among 7 phyla) were identified as potential parasites of skin mucus, gills mucus and intestine of 13 wild Mediterranean teleost fish species. Their distribution greatly differed among host species: 6 to 17 parasitic taxa were identified depending on the fish species (Table 2). In total, based on the 30 parasitic genera found, we listed 137 fish-parasite associations (Table 2). To our knowledge and according to the literature, 89 of these associations were new to the field of parasitology (see S2 Text). In most cases, the distribution of the different parasitic genera in the different fish tissues (skin mucus, gills mucus and intestine) is consistent with what has been described in the literature, for fungi, copepods, cnidarians, monogeneans, ciliates and most of the Apicomplexa. Some differences were nevertheless found for some parasitic taxa, in particular for nematodes and Digenea. As expected, the distribution of all monogenean genera in skin mucus and gills mucus is consistent with their life cycle [8,14]. Monogeneans are hermaphroditic and predominantly oviparous (including all genera identified here). The adults release eggs, from which ciliated larvae (oncomiracidium) hatch and swim freely. These larvae are attracted by fish mucus and colonize their skin [121–123]. Oncomiracidia then loose their ciliature and migrate from the skin to the gills of the host [122,124–125]. It is therefore not surprising to find monogeneans on both gills (adult organisms) and skin (larvae) of their sparid hosts.

Within the class Digenea, three genera, *Lecithochirium*, *Diphtherostomum* and *Cardiocephaloides*, differed slightly from previous studies. Indeed, *Cardiocephaloides* had only been identified previously in skin and gill mucus [84–85] and the two other genera were reported only in organs of the digestive system and it is not the case in our study. Several hypotheses can be formulated to explain this distribution. The digenean life cycle is complex and composed of different stages, typically with two intermediate hosts. When eggs hatch (released from adult worms present on the definitive host), they release free-swimming larvae (miracidia) that detect a first intermediate host and penetrate its tissues and skin [126–127]. Different life stages then develop in this host: sporocysts and/or rediae, which in turn release the cercarial stage, a free-swimming organism which infect the second intermediate host. It is therefore not surprising to find digenean parasites on the surface’s tissue of their hosts (skin and gills). Alternatively, the internal anatomy of teleost fishes could also have been a factor, particularly the position of the intestine which begins at the posterior edge of the gills and ends at the anus [128]. Due to its location, the intestine may have contaminated other organs, including the gills and skin.

While all parasitic genera belonging to the phylum Nematoda should be identified in or on the internal organs of their fish hosts, such as the intestine, some genera were identified on the skin mucus (*Acanthocheilus*, *Dichelyne*, *Cystidicola*, *Hysterothylacium* and *Aonchotheca*) and also on gills (*Contracaecum*, *Cucullanus*, *Cystidicola and Aonchotheca*). Among parasitic nematodes, some have an indirect life cycle, which means that they need several successive hosts to complete their life cycle and reach the adult stage [70,76,129]. This is the case for seven genera of nematodes found in the present study, identified on the gills or skin of fish (Table 3): the intermediate host is usually a crustacean (isopods, amphipods, copepods, decapods) and the final host is a teleost [69,76,130–134]. Sequences identified on the skin mucus or on gills may then correspond to larval stages or eggs of nematodes, contained in crustaceans that could have been on the fish's surfaces.

## Conclusion

In this study, we analyzed the eukaryotic sequences of a universal gene metabarcoding approach, initially targeting bacterial DNA, in order to identify potential endo- and ectoparasitic eukaryotic species associated with several wild Mediterranean teleost fish species. We illustrated the strong potential of this approach to reveal and identify an unexpectedly large spectrum of eukaryotic parasites within a host and therefore significantly increase our knowledge about the distribution of these organisms. The results obtained are consistent, for the most, with the literature, which highlights that data sets from metabarcoding approaches should therefore be used to the maximum, even beyond what they were originally intended for, as they can give us an overview of other communities in the studied environment. Thanks to this approach, we were able to detect a total of 30 parasitic genera distributed among 7 different phyla. This study also highlighted that each host has a different parasitic diversity. Despite its limitations, the metabarcoding approach provides a powerful, fast and readily available toolbox that could be applied for parasitological surveys. In general, DNA metabarcoding has an enormous potential to increase data acquisition in biodiversity surveys and therefore deliver key information to address many fundamental and applied research questions in ecology.

## Supporting information

S1 Text Shell script 18Sclean.sh. (DOCX)

S2 Text Fish-parasite associations known from the literature. (DOCX)

S1 Table Presence (black square) or absence (white square) of the eukaryotic parasitic taxa within fish teleost species with less than 10 reads. (DOCX)

S2 Table OTU table corresponding to water samples (negative controls at each sampling date). (XLSX)

S3 Table Distribution of different parasitic phyla within gills mucus, intestine and skin mucus. Each line represents the proportion of reads of the parasitic phyla in each tissue in relation to the number of total eukaryotic reads obtained for this taxa. The largest percentage for each phylum is in bold.
(DOCX)
