# Correction: Characterization of ecto- and endoparasite communities of wild Mediterranean teleosts by a metabarcoding approach

**DOI:** 10.1371/journal.pone.0223392

**Published:** 2019-09-26

**Authors:** The PLOS ONE Staff

There is an error in the caption for Table 2, “Presence (black square) or absence (white square) of eukaryotic parasitic taxa within fish teleost species”. The publisher apologizes for the error. Please see the complete, correct Table 2 caption here.

**Table 2:** Presence (black square) or absence (white square) of eukaryotic parasitic taxa within fish teleost species. The grey squares indicate fish-parasite associations already listed in the literature (S2 Text). References confirm the parasitic status of the genera. Fish species: ^1^*Diplodus annularis*, ^2^*Diplodus vulgaris*, ^3^*Gobius bucchichi*, ^4^*Gobius cruentatus*, ^5^*Gobius niger*, ^6^*Oblada melanura*, ^7^*Pagellus bogaraveo*, ^8^*Pagellus erythrinus*, ^9^*Sarpa salpa*, ^10^*Scorpaena notata*, ^11^*Serranus scriba*, ^12^*Spicara maena*, ^13^*Symphodus tinca*.

		Fish species												
		1	2	3	4	5	6	7	8	9	10	11	12	13
Phylum, Class	Genus													
Ascomycota, Eurotiomycetes	*Aspergillus* [55–56]													
Ascomycota, Dothideomycetes	*Cladosporium* [57]													
Arthropoda, Copepoda	*Caligus* [58–59]													
Arthropoda, Copepoda	*Chondracanthus* [60–61]													
Arthropoda, Copepoda	*Lepeophtheirus* [62–63]													
Arthropoda, Copepoda	*Taeniacanthus* [64–65]													
Cnidaria, Myxozoa	*Kudoa* [66]													
Cnidaria, Myxozoa	*Unicapsula* [67–68]													
Nematoda, Chromadorea	*Acanthocheilus* [69]													
Nematoda, Chromadorea	*Contracaecum* [70–71]													
Nematoda, Chromadorea	*Cucullanus* [18,72]													
Nematoda, Chromadorea	*Dichelyne* [17,73]													
Nematoda, Chromadorea	*Cystidicola* [74–75]													
Nematoda, Chromadorea	*Hysterothylacium* [76–77]													
Nematoda, Enoplea	*Aonchotheca* [78–79]													
Platyhelminthes, Monogenea	*Lamellodiscus* [16,80]													
Platyhelminthes, Monogenea	*Microcotyle* [81–82]													
Platyhelminthes, Monogenea	*Polylabris* [83–84]													
Platyhelminthes, Digenea	*Cardiocephaloides* [85–86]													
Platyhelminthes, Digenea	*Skoulekia* [87–88]													
Platyhelminthes, Digenea	*Accacoelium* [89]													
Platyhelminthes, Digenea	*Rhipidocotyle* [90–91]													
Platyhelminthes, Digenea	*Lecithochirium* [92–93]													
Platyhelminthes, Digenea	*Opisthorchis* [60,94]													
Platyhelminthes, Digenea	*Diphtherostomum* [95–96]													
Apicomplexa, Acanoidasida	*Theileria* [97–98]													
Apicomplexa, Conoidasida	*Cryptosporidium* [99–100]													
Apicomplexa, Conoidasida	*Eimeria* [101–102]													
Apicomplexa, Conoidasida	*Goussia* [103–104]													
Ciliophora, Oligohymenophorea	*Trichodina* [105–106]													
Dinoflagellata, Syndiniophyceae	Order Syndiniales [107]													
	**Total number of parasitic taxa**	**9**	**12**	**12**	**6**	**11**	**16**	**10**	**14**	**16**	**11**	**7**	**7**	**17**
