# Supplementary material for: Characterization of ecto- and endoparasite communities of wild Mediterranean teleosts by a metabarcoding approach
Source: PLoS One. 2019 Sep 10;14(9):e0221475. doi: 10.1371/journal.pone.0221475 (PMC6736230; doi:10.1371/journal.pone.0221475)
Supplement: S1 Text — (DOCX) [file pone.0221475.s001.docx]

#!/bin/bash

for j in $(ls *R1.fastq)

do i=${j%%_R1.fastq}

**#assemble reads with PEAR v.0.9.6**

pear -f ${i}_R1.fastq -r ${i}_R2.fastq -o pear.${i}

**#filter for quality**

usearch9 -fastq_filter pear.${i}.unassembled.forward.fastq -fastq_maxee 1.0 -fastaout pear.unmerged.r1.${i}.fas -fasta_cols 0

usearch9 -fastq_filter pear.${i}.unassembled.reverse.fastq -fastq_maxee 1.0 -fastaout pear.unmerged.r2.${i}.fas -fasta_cols 0

**#get names in forward read**

cat pear.unmerged.r1.${i}.fas | grep ">" > names_forward.${i}

**#get names in both**

gawk -v STUB=${i} 'BEGIN {RS=">"}FILENAME=="names_forward."STUB{a[$1]=$1}FILENAME=="pear.unmerged.r2."STUB".fas" {if(a[$1]){printf(">%s\n",$1)}}' names_forward.${i} pear.unmerged.r2.${i}.fas> names_both.${i}

**#get forward reads**

gawk -v STUB=${i} 'BEGIN {RS=">"}FILENAME=="names_both."STUB{a[$1]=$1}FILENAME=="pear.unmerged.r1."STUB".fas" {if(a[$1]){printf(">%s\n",$1);printf("%s\n",$3)}}' names_both.${i} pear.unmerged.r1.${i}.fas > pear.unmerged.qfiltered.r1.${i}.fas

**#get reverse reads**

gawk -v STUB=${i} 'BEGIN {RS=">"}FILENAME=="names_both."STUB{a[$1]=$1}FILENAME=="pear.unmerged.r2."STUB".fas" {if(a[$1]){printf(">%s\n",$1);printf("%s\n",$3)}}' names_both.${i} pear.unmerged.r2.${i}.fas > pear.unmerged.qfiltered.r2.${i}.fas

**#join both reads with an N**

paste pear.unmerged.qfiltered.r1.${i}.fas pear.unmerged.qfiltered.r2.${i}.fas | awk '{if($1~/>/){printf("%s\n",$1)}else{printf("%sN",$1);printf("%s\n",$2)}}' > combined.seq.${i}

**#change sequence names**

cat combined.seq.${i} | sed '1s/\>//' | gawk -v STUB=${i} 'BEGIN{RS=">"}{printf(">%s\n",STUB"."NR);printf("%s\n",$2)}' > ${i}_nb.fas

rm pear*

rm names*

done

**#merge sequences in a single file**

cat *_nb.fas > merged0.fas

**#renumber all sequences**

cat merged0.fas | sed '1s/\>//'| gawk 'BEGIN{RS=">"}{{sub(/\..*/,"."NR,$1)};printf(">%s\n",$1);printf("%s\n",$2)}'>merged.fasta

rm pear*

rm names*
