# Supplementary material for: Characterization of ecto- and endoparasite communities of wild Mediterranean teleosts by a metabarcoding approach
Source: PLoS One. 2019 Sep 10;14(9):e0221475. doi: 10.1371/journal.pone.0221475 (PMC6736230; doi:10.1371/journal.pone.0221475)
Supplement: S2 Text — (DOCX) [file pone.0221475.s002.docx]

| *Host species* | *Parasitic genus* | References |
| --- | --- | --- |
|  |  |  |
| *Diplodus annularis* | *Caligus* | Rosecchi 1985 |
|  | *Hysterothylacium* | Matic-Skoko *et al.,* 2004 |
|  | *Lamellodiscus* | Kaouachi *et al.,* 2012 |
|  | *Microcotyle* | Noisy and Maillard, 1980 |
|  | *Diphtherostomum* | Radujkovic and Raibaut, 1989 |
|  |  |  |
|  |  |  |
| *Diplodus vulgaris* | *Contracaecum* | Papoutsoglou 1976 |
|  | *Lamellodiscus* | Kaouachi *et al.,* 2012 |
|  | *Skoulekia* | Palacios-Abella *et al.,* 2017 |
|  | *Diphtherostomum* | Radujkovic and Raibaut, 1989 |
|  | *Eimeria* | Radujkovic and Raibaut, 1989 |
|  |  |  |
|  |  |  |
| *Gobius bucchichi* | *Cucullanus* | Sasal *et al.,* 1996 |
|  |  |  |
|  |  |  |
| *Gobius cruentatus* | *Lecithochirium* | Bartoli *et al.,* 2005 |
|  |  |  |
|  |  |  |
| *Gobius niger* | *Dichelyne* | Pronkina *et al.,* 2017 |
|  | *Hysterothylacium* | Radujkovic and Raibaut, 1989 |
|  | *Aonchotheca* | Sezgin *et al.,* 2017 |
|  | *Lecithochirium* | Paradiznik and Radujkovic, 2007 |
|  | *Trichodina* | Zander 2004 |
|  |  |  |
|  |  |  |
| *Oblada melanura* | *Cucullanus* | Öktener 2014 |
|  | *Dichelyne* | Radujkovic and Raibaut, 1989 |
|  | *Hysterothylacium* | Radujkovic and Raibaut, 1989 |
|  | *Lamellodiscus* | Desdevises *et al.,* 2002 |
|  | *Cardiocephaloides* | Born-Torrijos *et al*, 2016 |
|  | *Diphtherostomum* | Papoutsoglou 1976 |
|  |  |  |
|  |  |  |
| *Pagellus bogaraveo* | *Contracaecum* | Hermida and Mota, 2012 |
|  | *Cucullanus* | Moravec and Justine, 2018 |
|  | *Lamellodiscus* | Desdevises *et al.,* 2000 |
|  |  |  |
|  |  |  |
| *Pagellus erythrinus* | *Caligus* | Özak *et al.,* 2018 |
|  | *Contracaecum* | Papoutsoglou 1976 |
|  | *Cucullanus* | Ternengo *et al.,* 2009 |
|  | *Dichelyne* | Nawal and Sabiha, 2016 |
|  | *Hysterothylacium* | Ternengo *et al.,*2009 |
|  | *Cardiocephaloides* | Born-Torrijos *et al*, 2016 |
|  | *Skoulekia* | Palacios-Abella *et al.,* 2017 |
|  | *Eimeria* | Radujkovic and Raibaut, 1989 |
|  |  |  |
|  |  |  |
| *Sarpa salpa* | *Caligus* | Glober *et al.,* 2002 |
|  | *Lamellodiscus* | Strona *et al.,* 2010 |
|  |  |  |
|  |  |  |
| *Scorpaena notata* | *Hysterothylacium* | Radujkovic and Raibaut, 1989 |
|  | *Eimeria* | Lom and Dyková 1995 |
|  |  |  |
|  |  |  |
| *Serranus scriba* | *Eimeria* | Radujkovic and Raibaut, 1989 |
|  |  |  |
|  |  |  |
| *Spicara maena* | *Lamellodiscus* | Desdevises *et al.,* 2002 |
|  | *Accacoelium* | Ahuir-Baraja *et al.,* 2015 |
|  |  |  |
|  |  |  |
| *Symphodus tinca* | *Caligus* | Benmansour and Ben Hassen, 1997 |
|  | *Cucullanus* | Öktener 2014 |
|  | *Hysterothylacium* | Ternengo *et al.,* 2009 |
|  | *Aonchotheca* | Muñoz and Díaz, 2015 |
|  | *Lecithochirium* | Radujkovic and Sundic, 2014 |
|  | *Eimeria* | Daoudi *et al.,* 1989 |
|  | *Goussia* | Sitjà-Bobadilla *et al.,* 1996 |

References:

Ahuir-Baraja AE, Padrós F, Palacios-Abella JF, Raga JA, Montero FE. *Accacoelium contortum* (Trematoda: Accacoeliidae) a trematode living as a monogenean: Morphological and pathological implications. Parasites and Vectors. 2015;8(1):540.

Bartoli P, Gibson D, Bray R. Digenean species diversity in teleost fish from a nature reserve off Corsica, France (Western Mediterranean), and a comparison with other Mediterranean regions. Journal of Natural History. 2005;39(1):47–70.

Benmansour B, Ben Hassine OK. Première mention en Tunisie de certains Caligidae et Lernaeopodidae (Copepoda), parasites de poissons Teleostéens. In: Ichtyophysiologica Acta, Tome 20. 1997. p. 157–75.

Born-Torrijos A, Poulin R, Pérez-del-Olmo A, Culurgioni J, Raga JA, Holzer AS. An optimised multi-host trematode life cycle: fishery discards enhance trophic parasite transmission to scavenging birds. International Journal for Parasitology. 2016;46(11):745–53.

Daoudi F, Radujkovic B, Marques A, Bouix G. Nouvelles espèces de Coccidies (Apicomplexa, Eimeriidae) des genres *Eimeria* Schneider, 1875 et *Epieimeria* Dykova et Lom, 1981, parasites des poissons marins méditerranéens. Bulletin du Museum National d’Histoire Naturelle Paris. 1989;4:743–53.

Desdevises Y, Jovelin R, Jousson O, Morand S. Comparison of ribosomal DNA sequences of *Lamellodiscus* spp. (Monogenea, Diplectanidae) parasitising *Pagellus* (Sparidae, Teleostei) in the North Mediterranean Sea: Species divergence and coevolutionary interactions. International Journal for Parasitology. 2000;30(6):741–6.

Desdevises Y, Morand S, Jousson O, Legendre P. Coevolution between *Lamellodiscus* (Monogenea: Diplectanidae ) and Sparidae (Teleostei ): The Study of a Complex Host-Parasite System. Evolution. 2002;56(12):2459–71

Desdevises Y, Morand S, Legendre P. Evolution and determinants of host species city in the genus *Lamellodiscus* (Monogenea). Biological Journal of the Linnean Society. 2002;77:431–43.

Grobler JN, Van As GJ, Olivier ASP. Description of the previously unknown male of *Caligus mortis* Kensley, 1970 (Copepoda: Caligidae), parasite of intertidal fish from South Africa. Folia Parasitologica. 2002;49(2):131–6.

Hermida M, Mota R. Infection levels and diversity of anisakid nematodes in blackspot seabream, *Pagellus bogaraveo*, from Portuguese waters. Parasitology Research. 2012;110:1919–28.

Kaouachi N, Chahinez B, Bensouilah M, Quilichini Y. Les Monogènes parasites du genre *Diplodus* dans l’Est du littoral algérien. Bulletin de l’Institut Scientifique. 2012;34(1):57–63.

Lom J, Dyková I. Studies on protozoan parasites of Australian fishes. Notes on coccidian parasites with description of three new species. Systematic Parasitology. 1995 Jun;31(2):147–56.

Matic-Skoko S, Antolić B, Kraljevic M. Ontogenetic and seasonal feeding habits of the annular seabream (*Diplodus annularis* L.) in Zostera sp. beds, eastern Adriatic Sea. Journal of Applied Ichthyology. 2004;20:376–81.

Moravec F, Justine J. Three new species of *Cucullanus* (Nematoda : Cucullanidae ) from marine fishes off New Caledonia, with a key to species of *Cucullanus* from Anguilliformes. Parasite. 2018;25:51.

Muñoz G, Díaz PE. Checklist of parasites of labrid fishes (Pisces: Labridae). 2015. 95 pp.

Nawal M, Sabiha M. Contribution à l’étude des Nématodes des poissons téléostéens du Golfe de Bejaia. 2016.

Noisy D, Maillard C. Microhabitat branchial préférentiel de *Microcotyle chrysophrii*. Annales de Parasitologie Humaine et Comparée. 1980;55(1):33–40.

Öktener A. An Updated Checklist Of Parasitic Helminths Of Marine Fish From Turkey. Transylvanian Review of Systematical and Ecological Research. 2014;16.

Özak AA, Ternengo S, Boxshall A. Redescription *of Caligus pagelli* Delamare Deboutteville & Nunes-Ruivo, 1958 (Copepoda : Caligidae) on the common pandora, *Pagellus erythrinus* (Linnaeus), in western Mediterranean waters off Corsica, France. Systematic Parasitology. 2018;95(6):591–601.

Palacios-Abella JF, Georgieva S, Mele S, Raga JA, Isbert W, Kostadinova A, et al. *Skoulekia erythrini* n. sp. (Digenea: Aporocotylidae): a parasite of *Pagellus erythrinus* (L.) (Perciformes: Sparidae) from the western Mediterranean with an amendment of the generic diagnosis. Systematic Parasitology. 2017;94(6):669–88.

Papoutsoglou, SE. Metazoan parasites of fishes from Saronicos Gulf, Athens, Greece. 1976.

Paradiznik V, Radujkovic B. Digenea trematodes in fish of the North Adriatic Sea. Acta Adriatica. 2007;48(2):115–29.

Pronkina NV, Dmitrieva EV, Polyakova TA, Popyuk MP. The Lifecycle of *Dichelyne minutus* (Rudolphi, 1819) (Nematoda: Cucullanidae) in the Estuarine Biocenosis of the Black Sea. Parasitology. 2017;43(2):148–55.

Radujkovic B, Raibaut A. Parasites des poissons marins du Montenegro: liste des espèces de poissons avec leurs parasites. Acta Adriatica. 1989;30(1/2):307–19.

Radujkovic B, Sundic D. Parasitic flatworms (Platyhelminthes: Monogenea, Digenea, Cestoda) of fishes from the Adriatic Sea. Natura Montenegrina. 2014;13(1):7–280.

Rosecchi E. L’alimentation de *Diplodus annularis*, *Diplodus sargus, Diplodus vulgaris et Sparus aurata* (Pisces, Sparidae) dans le Golfe du Lion et les lagunes littorales. Revue des travaux de l’Institut des pêches maritimes. 1985;49:125–41.

Sasal P, Faliex E, Morand S. Parasitism of *Gobius bucchichii* Steindachner, 1870 (Teleostei, Gobiidae) in protected and unprotected marine environments. Journal of Wildlife Diseases. 1996;32(4):607–13.

Sezgin M, Bat L, Ürkmez D, Arıcı E, Oztürk B. Black Sea Marine Environment: The Turkish Shelf. 2017.

Sitjà-Bobadilla A, Palenzuela O, Alvarez-Pellitero P. Light microscopic description of *Eimeria sparis* sp nov and *Goussia sparis* sp nov (Protozoa: Apicomplexa) from *Sparus aurata* L (Pisces: Teleostei). Parasitology research. 1996;82:323–32.

Strona G, Stefani F, Galli P. Monogenoidean parasites of Italian marine fish: An updated checklist. Italian Journal of Zoology. 2010;77(4):419–37.

Ternengo S, Levron C, Mouillot D, Marchand B. Site influence in parasite distribution from fishes of the Bonifacio Strait Marine Reserve (Corsica Island, Mediterranean Sea). Parasitology Research. 2009;104:1279–87.

Zander CD. Four-year monitoring of parasite communities in gobiid fishes of the south-western Baltic. Parasitology Research. 2004;93:17–29.
