# Supplementary material for: Characterization of ecto- and endoparasite communities of wild Mediterranean teleosts by a metabarcoding approach
Source: PLoS One. 2019 Sep 10;14(9):e0221475. doi: 10.1371/journal.pone.0221475 (PMC6736230; doi:10.1371/journal.pone.0221475)
Supplement: S1 Table — (DOCX) [file pone.0221475.s003.docx]

|  |  | Fish species | | | | | | | | | | | | |
| --- | --- | --- | --- | --- | --- | --- | --- | --- | --- | --- | --- | --- | --- | --- |
|  |  | *Diplodus annularis* | *Diplodus vulgaris* | *Gobius bucchichi* | *Gobius cruentatus* | *Gobius niger* | *Oblada melanura* | *Pagellus bogaraveo* | *Pagellus erythrinus* | *Sarpa salpa* | *Scorpaena notata* | *Serranus scriba* | *Spicara maena* | *Symphodus tinca* |
| Phylum, Class | Genus |  |  |  |  |  |  |  |  |  |  |  |  |  |
| Ascomycota, Dothideomycetes | *Hortaea* |  |  |  |  |  |  |  |  |  |  |  |  |  |
| Ascomycota, Dothideomycetes | *Alternaria* |  |  |  |  |  |  |  |  |  |  |  |  |  |
| Ascomycota, Saccharomycetes | *Metschnikowia* |  |  |  |  |  |  |  |  |  |  |  |  |  |
| Cnidaria, Myxozoa | *Ortholinea* |  |  |  |  |  |  |  |  |  |  |  |  |  |
| Platyhelminthes, Digenea | *Mesometra* |  |  |  |  |  |  |  |  |  |  |  |  |  |
