# Supplementary material for: Characterization of ecto- and endoparasite communities of wild Mediterranean teleosts by a metabarcoding approach
Source: PLoS One. 2019 Sep 10;14(9):e0221475. doi: 10.1371/journal.pone.0221475 (PMC6736230; doi:10.1371/journal.pone.0221475)
Supplement: S3 Table — Each line represents the proportion of reads of the parasitic phyla in each tissue in relation to the number of total eukaryotic reads obtained for this taxa. The largest percentage for each phylum is in bold. (DOCX) [file pone.0221475.s005.docx]

|  | Location (%) | | |
| --- | --- | --- | --- |
|  | Gills mucus | Skin mucus | Intestine |
| Ascomycota | 34.5 | **65.5** | 0 |
| Arthropoda, Copepoda | 15.3 | **82.1** | 2.7 |
| Cnidaria | 2.3 | **93.9** | 3.7 |
| Nematoda | 2.7 | 11.5 | **85.7** |
| Platyhelminthes, Monogenea | **62.9** | 37.1 | 0 |
| Platyhelminthes, Digenea | **90.9** | 2.4 | 6.6 |
| Apicomplexa | 3.6 | 18.6 | **77.8** |
| Ciliophora | **98.7** | 1.3 | 0 |
| Dinoflagellata | 7.9 | **91.6** | 0.4 |
